# Proactive consultation-liaison-services (CL): comparing PHQ-4 screening with traditional referral pathways in medical inpatients – a prospective comparative pilot study

**DOI:** 10.1186/s12888-026-08222-7

**Published:** 2026-06-15

**Authors:** Paul Köbler, Linda Wilfert, Alexander Dechêne, Eva K. Krauß-Köstler, Christiane Waller, Barbara Stein

**Affiliations:** 1 Department of Psychosomatic Medicine and Psychotherapy, Paracelsus Medical University, Nuremberg General Hospital, Prof.-Ernst-Nathan-Str. 1, 90419 Nuremberg, Germany; 2 Department of Internal Medicine 6, Gastroenterology/Hepatology/Endocrinology, Paracelsus Medical University, Nuremberg General Hospital, Nuremberg, Germany

**Keywords:** Screening, Depression, Anxiety, Hospitalization, Consultation-liaison, Diagnosis, Inpatients

## Abstract

**Background:**

Psychiatric comorbidities are highly prevalent among general hospital inpatients but often remain undetected when mental health referrals rely solely on clinical judgment. Systematic screening may improve identification, yet comparative evidence using structured diagnostic interviews remains limited. The study evaluates diagnostic confirmation rate of systematic PHQ-4 screening versus clinician-initiated referral in identifying psychiatric comorbidities among medical inpatients.

**Methods:**

In this quasi-experimental pilot study, systematic screening using the Patient Health Questionnaire-4 (PHQ-4) was implemented on one medical ward (*n* = 127 admissions), while two comparable wards followed treatment as usual (TAU; *n* = 267 admissions), with psychosomatic consultations initiated by medical staff based on clinical judgment. Patients screening positive (PHQ-4 ≥ 6 or subscale ≥ 4) were referred for consultation. All referred patients underwent structured diagnostic assessment using the Mini-DIPS interview. Primary outcomes were the proportion of referred patients with a confirmed psychiatric diagnosis (diagnostic confirmation rate) and overall identification rates. Secondary outcomes included time from admission to referral and consultation, as well as treatment recommendations and their uptake.

**Results:**

Among interviewed patients, screening was associated with a numerically higher rate of diagnostic confirmation than TAU (11/13, 84.6% vs. 8/14, 57.1%), although this difference did not reach statistical significance. A higher proportion of admissions with confirmed psychiatric disorders was observed in the screening group (11/127, 8.7%) compared with TAU (8/267, 3.0%). Referral rates were higher in the screening group (21/127, 16.5% vs. 22/267, 8.2%), and time to consultation was shorter (median 1 vs. 3.5 days). Depressive and anxiety disorders predominated. Follow-up consultation showed high adherence, whereas uptake of inpatient treatment was low.

**Conclusions:**

Systematic PHQ-4 screening was associated with higher detection of psychiatric comorbidities and earlier consultation-liaison involvement, suggesting that clinician-initiated referral alone may miss substantial psychiatric morbidity. However, given the quasi-experimental design and limited sample size, the findings should be interpreted cautiously. Larger randomized studies are needed to evaluate implementation and patient outcomes.

## Background

Psychiatric comorbidities are common among general hospital inpatients but frequently remain undetected or untreated. The prevalence of depression in general medical and surgical wards ranges from 5% to 34%, with a pooled mean of 12% (95% prediction interval: 4–32%) [1]. Anxiety disorders are similarly prevalent, with a pooled point prevalence of 8% across hospitalized populations [2].

Despite these figures, detection rates remain low [3, 4]. Traditional consultation-liaison (C-L) services, which rely on clinician referrals, often address only a fraction of actual psychiatric care needs [5]. Moreover, diagnoses based solely on clinical judgment—without standardized tools—show only moderate agreement with diagnostic gold standards, suggesting a substantial risk of misclassification [6, 7].

Undetected psychiatric disorders are associated with adverse outcomes, including worsened trajectories of physical illness, increased mortality, and reductions in quality-adjusted life years [8–10]. Mental health conditions also elevate healthcare costs and reduce adherence to medical treatment [7, 11, 12]. While systematic early detection and timely intervention may improve patient outcomes and potentially reduce preventable readmissions or the need for intensive measures (e.g., constant observation in suicide risk cases), this remains a topic of ongoing investigation [5, 9]. Studies implementing automated screening procedures in acute medical settings such as screening with wordlists in the Electronic Medical Records have reported up to a threefold increase in psychiatric consultations, suggesting that unmet needs are being uncovered [5]. Clinicians also report that these tools help them identify at-risk patients they might otherwise overlook [11, 13]. Patient involvement in the screening process appears to further enhance its impact. For example, structured feedback provided to both patients and their general practitioners has been associated with improved outcomes and better communication [14, 15].

Validated self-report instruments offer a promising avenue to improve the identification of common mental disorders in medical settings. Several screening instruments have been proposed to improve detection of common mental disorders in medical settings, including questionnaires such as the Patient Health Questionnaire-9 (PHQ-9), the Generalized Anxiety Disorder-7 (GAD-7) [14, 15], the Geriatric Depression Scale (GDS) or the General Health Questionnaire (GHQ) [16]. While these instruments demonstrate good psychometric properties, their implementation in routine hospital workflows may be limited by time constraints and integration into clinical practice. The Patient Health Questionnaire-4 (PHQ-4) is an ultra-brief screening tool with strong psychometric properties for detecting symptoms of depression and anxiety and offers a pragmatic alternative for rapid screening in busy inpatient settings [17].

Most previous studies have used screening instruments primarily to identify participants for subsequent intervention trials or to evaluate treatment outcomes following screening. Trials such as DEPSCREEN-INFO [14] and GET.FEEDBACK.GP [15], for example, focused on the effects of feedback or treatment after screening rather than on the identification process itself. Consequently, the fundamental question of whether systematic screening improves the initial identification of psychiatric disorders compared with routine clinician-initiated referral remains insufficiently addressed.

Systematic reviews also highlight methodological limitations in the existing literature, including the exclusion of patients with more severe psychiatric illness and a lack of proactive or integrated identification approaches [16].

A meta-analysis of 38 RCTs on C-L interventions found small to moderate effects on depressive and anxiety symptoms, with integrated collaborative care models showing a modest but significant impact on depression [18]. However, most included studies lacked structured diagnostic interviews to confirm psychiatric diagnoses, limiting conclusions about the diagnostic utility of different identification strategies.

Because a positive screening result does not constitute a clinical diagnosis and requires further clinical assessment [4], structured diagnostic interviews represent an important reference standard when evaluating identification strategies.

Together, these limitations highlight a gap in the literature: to our knowledge, no studies have directly compared screening-based identification with routine clinician referral using structured diagnostic interviews as a reference standard.

### Rationale for the present study

This pilot study addresses a critical gap by directly comparing systematic screening using the PHQ-4 with routine identification by medical staff in terms of diagnostic confirmation rate. We examined referred patients in two non-randomized groups—screening vs. usual care (no systematic screening, referral by medical staff)—and used structured clinical interviews (MINI-DIPS) as the reference standard. Although the study does not aim to estimate psychiatric prevalence, it explores differences in case identification within the referral process. The primary outcome was the proportion of referred patients with confirmed psychiatric diagnoses. Secondary outcomes included time from admission to referral, time from admission to consultation, and uptake of clinical treatment recommendations.

## Methods

This study is reported in accordance with the Strengthening the Reporting of Observational Studies in Epidemiology (STROBE) statement [19]. STROBE provides essential items for transparent reporting of observational research, ensuring sufficient methodological detail for critical appraisal and replication. Given the non-randomized, observational design of this pilot investigation, the STROBE framework was selected to promote methodological rigor and comparability with similar studies.

### Mental health consultation services

At the study site, mental health care for medical inpatients is provided through two separate consultation services. This reflects the structure of the German healthcare system, in which psychiatry and psychosomatic medicine are distinct medical specialties with partly overlapping but different clinical responsibilities.

Psychosomatic consultation service. The Department of Psychosomatic Medicine and Psychotherapy provides consultation-liaison services primarily for patients with anxiety disorders, depressive disorders, somatoform disorders, adjustment and stress-related disorders, and trauma-related conditions. This service was the focus of the present investigation.

Psychiatric consultation service. The Department of Psychiatry operates independently and primarily manages severe mental disorders including psychotic disorders, bipolar disorder, and acute psychiatric crises. Psychiatric consultations occurred during the study period in both groups and are reported descriptively. However, as the psychiatric consultations did not employ standardized diagnostic procedures, they were not included in primary outcome analyses, and their diagnostic precision could not be evaluated within this study framework.

Throughout this manuscript, we use the term “psychiatric comorbidities” to refer to all mental health conditions diagnosed according to standardized classification systems (ICD-10), regardless of which consultation service provided treatment.

### Study design and setting

We conducted a naturalistic, two-group comparative study over a six-week period from November to December 2024 at the Department of Internal Medicine, Gastroenterology, Paracelsus Medical University, Nuremberg General Hospital, a large tertiary care university hospital in Germany with approximately 2,200 inpatient beds and around 335,000 inpatient and outpatient cases treated annually across two sites. The study included all newly admitted inpatients across three medical wards with comparable monthly admission volumes and similar somatic treatment profiles.

### Study groups

#### Intervention group (screening group)

Patients admitted to one designated ward formed the intervention group. Trained members of the study group (PK and LW) visited this ward each weekday to approach all newly admitted patients. Using a standardized script, they introduced the study and administered the Patient Health Questionnaire-4 (PHQ-4), a brief self-report screening tool for anxiety and depression. Completed forms were collected and scored immediately.

If the total PHQ-4 score met or exceeded the threshold of ≥ 6, or if either subscale (PHQ-2 depression or PHQ-2 anxiety) scored ≥ 4, a structured clinical diagnostic interview—the Mini-DIPS (*Short Diagnostic Interview for Mental Disorders: Diagnostisches Kurzinterview bei Psychischen Störungen*)—was conducted to confirm a psychiatric diagnosis by PK or LW. These cut-offs follow established PHQ-4 scoring recommendations and have also been applied in hospital-based somatic patient populations undergoing psychosomatic consultation screening [4]. Further psychosomatic consultation and follow-up care were provided as clinically indicated.

#### Control group (treatment as usual, TAU)

Patients admitted to two other wards formed the control group. These wards continued standard care. In this condition, psychosomatic consultations were initiated only by treating physicians and nursing staff based on clinical judgment. At the initial CL consultation, the same structured diagnostic interview (Mini-DIPS) was used to assess psychiatric comorbidity by PK or LW. Further psychosomatic care followed the same clinical protocols as the intervention group.

### Follow-up

For patients who received a psychiatric diagnosis and treatment recommendation during the index hospital stay, we assessed whether they had accessed the recommended care at three months. For hospital-based services (continued consultation-liaison follow-up or specialized inpatient psychosomatic care), participation was verified through the hospital’s internal electronical documentation system. For external referrals to outpatient providers or counselling centers, patients were contacted directly to confirm attendance at recommended services.

### Sample

As this was a pilot study, we did not conduct a formal power calculation. The sample size was determined pragmatically based on the number of new admissions over a six-week observation period, with the primary aim of exploring sample characteristics and preliminary differences between groups.

Patients were excluded from screening if the questionnaire was not available in any language they spoke, or if they lacked sufficient proficiency in German to participate in the diagnostic interview (Mini-DIPS). Additional exclusion criteria included terminal physical illness, severe cognitive impairment that precluded reliable participation in a structured diagnostic interview (delirium or advanced dementia), or other acute medical conditions preventing meaningful study participation. The minimum age for inclusion was 18 years.

### Instruments

#### Patient health questionnaire-4 (PHQ-4)

The PHQ-4 is a validated ultra-brief screening instrument consisting of four items that assess core symptoms of depression and anxiety on a 4-point Likert scale (0 = not at all to 3 = nearly every day) [17, 20]. The tool demonstrates good psychometric properties and is well-suited for use in general medical inpatient settings [4].

To support inclusive application, the questionnaire was available in translation for 13 additional languages: Amharic, Arabic, Czech, Danish, English, French, Indonesian, Italian, Polish, Portuguese, Russian, Turkish, and Ukrainian.

#### Short diagnostic interview for mental disorders (Mini-DIPS)

The Mini-DIPS is a standardized clinical interview designed for rapid yet reliable assessment of psychiatric disorders according to ICD-10 criteria [21]. The interview takes approximately 30 min to complete and has been validated for use in both clinical and research contexts. Interviewer 1 (PK) was a clinical practitioner with ten years of professional experience in psychotherapy and had trained and supervised interviewer 2 (LW) in the application of the Mini-DIPS, who was in her second year of practice. Prior to study initiation, the interviewers underwent structured training in the use of the Mini-DIPS, including familiarization with the interview protocol, supervised observation of diagnostic interviews, and supervised practice interviews with patients, followed by joint case discussion and feedback.

### Variables and data collection

Individual patient characteristics.

- Sociodemographic characteristics.
- Main somatic diagnoses (based on hospital records).
- Psychiatric comorbidities (based on Mini-DIPS interview).

Structural features of the ward.

- Number of screenings conducted.
- Number of psychosomatic consultations requested.
- Number of psychiatric consultations requested.
- Number of consultations completed.
- Consultation rate (percentage of all admitted patients who received a consultation).
- Completion rate (percentage of requested consultations actually conducted).
- Length of stay in somatic inpatient treatment.

Service Delivery Indicators.

- Lag time to referral for consultation (relative to admission).
- Lag time to consultation execution (relative to admission).
- Type of follow-up psychosomatic care or clinical recommendation provided.
- Follow-up data on patients’ access to the recommended care three months after the initial consultation.

### Data calculation and missing data

An available case analysis was employed to address missing data. No imputation methods were applied. For each outcome variable, only participants with complete data at the corresponding assessment points were included in the analysis, resulting in varying sample sizes across statistical tests. We used IBM SPSS Statistics (version 28) for statistical analyses. Descriptive statistics were calculated. Given the exploratory nature of this pilot study, inferential statistical tests were applied to key comparisons between groups, including referral rates, lag times, and diagnostic confirmation rates. These analyses should be interpreted cautiously due to the limited sample size. Differences in proportions were assessed using Fisher’s exact test, and differences in continuous variables were analyzed using the Mann–Whitney U test.

## Results

### Patient flow through screening and referral pathways

During the study period, 127 patients were admitted to the intervention ward, of whom 104 completed PHQ-4 screening. Twenty-one patients screened above the clinical cutoff and were referred for psychosomatic consultation. Structured diagnostic interviews (Mini-DIPS) were completed in 13 of these patients.

In the control wards, 271 patients were admitted during the same period. Medical staff initiated 22 psychosomatic consultations based on clinical judgment, of which 14 patients received a diagnostic interview.

**Fig. 1:**
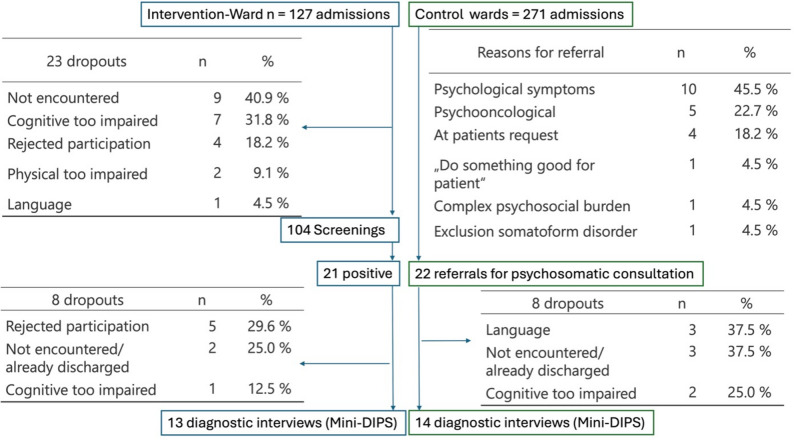
Flow of participants through the study. Note The intervention ward (*n* = 127 admissions) underwent systematic screening with the PHQ-4, resulting in 104 completed screenings and 21 positive cases referred for psychosomatic consultation. The control wards (*n* = 271 admissions) followed treatment-as-usual (TAU) with referrals initiated by medical staff (*n* = 22). Reasons for referral in the TAU group are listed on the right. Numbers and percentages of dropouts before the diagnostic interview are shown for both groups. Final numbers of completed structured clinical interviews (Mini-DIPS) are indicated at the bottom

The complete patient flow, including reasons for referral and reasons for non-completion of diagnostic interviews, is presented in Fig. 1. Non-interviewed patients on the intervention ward were significantly older than interviewed patients (mean age 75.1 vs. 57.4 years, *p* = 0.04), while screening scores, sex, and length of stay did not differ. No significant differences in sociodemographic or clinical variables were observed between interviewed and non-interviewed patients in the control group.

### Baseline characteristics of the study population

Table 1 presents the sociodemographic characteristics, treatment-related variables, and primary somatic diagnoses of participants in both study groups. The intervention group (*n* = 104) and control group (*n* = 271) did not differ significantly in age, sex distribution, or length of stay. Similarly, no significant sociodemographic differences were observed within either study arm—neither between patients who screened positive (*n* = 21) or negative (*n* = 83) on the intervention ward, nor between patients who were referred for consultation (*n* = 22) or not referred (*n* = 249) on the control wards. However, patients referred for psychosomatic consultation on control wards had a markedly longer mean hospital stay (16.7 days) compared with those who screened positive (8.0 days), screened negative (6.6 days), or were not referred (5.5 days). Two statistical outliers with very long admissions drove this difference. When calculated as medians, referred patients still had the longest stays (9 days) compared with screened-positive (7 days), screened-negative (4 days), and non-referred patients (3 days).

**Table 1:** Demographic characteristics, treatment data, and somatic main diagnoses for the intervention group and control group, as well as for the four subgroups (screened positive, screened negative, referred for consultation, not referred for consultation). SD = Standard deviation

	**Intervention group** **(n = 104)**		**Control group** **(n = 271)**	
**Demographics**				
Female (%)	57	(54.8%)	149	(55.0%)
Age, years (mean [SD])	64.1	[18.7]	64.3	[17.4]
Age, years (range)	19–94		18–95	
	*Screened positive* (*n* = 21)	*Screened negative* (*n* = 83)	*Referral f. Cons.* (*n* = 22)	*No Referral. f. Cons.* (*n* = 249)
% of sample	20.2%	79.8%	8.1%	91.9%
Female (%)	13 (61.9%)	44 (53.0%)	14 (63.6%)	138 (55.4%)
Age, years (mean [SD])	64.1 [19.7]	64.0 [18.6]	67.1 [16.7]	64.2 [17.5]
Age, years (range)	19–89	20–94	29–89	18–95
	**Intervention group** **(n = 104)**		**Control group** **(n = 271)**	
**Treatment data**				
Length of stay, days (mean [SD])	6.8	[7.6]	6.3	[9.6]
Length of stay, days (median [range])	5	[1–50]	3	[1–86]
Psychiatric referral in addition (%)	3	(2.9%)	12	(4.4%)
	*Screened positive* (*n* = 21)	*Screened negative* (*n* = 83)	*Referral f. Cons.* (*n* = 22)	*No Referral. f. Cons.* (*n* = 249)
Length of stay, days (mean [SD])	8.0 [7.6]	6.6 [7.7]	16.7 [23.4]	5.5 [6.9]
Length of stay, days (median [range])	7 [3–37]	4 [1–50]	9 [1–86]	3 [1–47]
Psychiatric referral in addition (%)	2 (9.5%)	1 (1.2%)	4 (18.2%)	11 (4.4%)
	Screened positive and interviewed (n = 13)		Referred f. Consultation and interviewed (n = 14)	
Lag time admission and referral for consultation (mean [SD])	1.7	[1.2]	7.6	[16.1]
Lag time admission and referral for consultation (median [range])	1.0	[1–5]	2.5	[0–62]
Lag time admission and consultation (mean [SD])	1.9	[1.3]	8.6	[16.1]
Lag time admission and consultation (median [range])	1.0	[1–5]	3.5	[0–63]
	**Intervention group** **(n = 104)**		**Control group (n = 271)**	
**Somatic Main diagnoses**				
**Diseases of the Digestive System** *Liver cirrhosis*,* gallstone disease*,* acute/chronic pancreatitis*,* gastric and duodenal ulcers*,* gastroenteritis and enteritis*, etc.	53	(51.0%)	153	(56.5%)
**Malignant Neoplasms** *(Liver/intrahepatic*,* pancreas*,* colon*,* rectum*,* esophagus*,* bladder*,* etc.)*	5	(4.8%)	24	(8.9%)
**Neoplams-in situ/benign/uncertain** *(Benign neoplasms (colon*,* rectum*,* appendix*,* stomach etc.)*,* neoplasms of uncertain/unknown behavior*,* etc.)*	6	(5.8%)	29	(10.7%)
**Diseases of the Circulatory System** *(Hypertension*,* Secondary right heart failure*,* Stroke*,* Artherosclerosis*,* Thrombosis*,* etc.)*	7	(6.7%)	7	(2.6%)
**Endocrine**,** Nutritional**,** and Metabolic Diseases** *(Diabetes mellitus*,* Cachexia*,* Addisonian crisis)*	2	(1.9%)	4	(1.5%)
**Mental and Behavioral Disorders** *(Alcohol abuse*,* severe depressie episode*,* somatoform disorder)*	1	(1.0%)	6	(2.2%)
**Diseases of the Respiratory System** *(Pneumonia*,* COPD*,* Asthma*,* Interstitial pulmonoray disease*,* etc.)*	11	(10.6%)	14	(5.2%)
**Diseases of the Genitourinary System** *(Urinary tract infection*,* Kidney failure)*	1	(1.0%)	6	(2.2%)
**Diseases of the Blood** *(Anemia)*	4	(3.8%)	5	(1.8%)
**Musculoskeletal Disorders** *(Radiculopathy*,* Polyarthritis*,* Myalgia*,* Fractures*,* etc.)*	4	(3.8%)	4	(1.5%)
**Infectious diseases** *(Mononucleosis*,* Bacterial infections*,* Sepsis)*	4	(3.8%)	4	(1.5%)
**Symptoms**,** Signs**,** and Abnormal Findings**,** Postprocedural and Mechanical Complications**,** Other / Unspecified Conditions or other diseases**	6	(5.8%)	15	(5.5%)

Primary treatment diagnoses were broadly comparable between intervention and control wards, with diseases of the digestive system representing the most frequent category in both groups (Table 1). Differences in diagnostic distribution were small, and none reached statistical significance.

### Psychiatric diagnoses and subsequent treatment recommendations

Among the 27 patients who underwent the structured clinical interview (Mini-DIPS), 19 (70.4%) received at least one psychiatric diagnosis. Detailed diagnostic frequencies are presented in Table 2.

**Table 2:** Frequencies of validated psychiatric diagnoses as assessed by the structured clinical interview (Mini-DIPS). PTSD: Posttraumatic Stress Disorder; GAD: Generalized Anxiety Disorder

*n* = 27 interviews	*n*	%
Affective disorders	12	(44.4%)
Bipolar II	1	(3.7%)
Depression (F32.0-F33.2)	11	(40.1%)
Alcohol: Dependence syndrom	4	(14.8%)
Reaction to severe stress, and adjustment disorders	3	(11.1%)
PTSD	2	(7.4%)
Adjustment disorder	1	(3.7%)
Anxiety disorders	3	(11.1%)
Social phobia	1	(3.7%)
Specific phobia	1	(3.7%)
GAD	1	(3.7%)
Somatoform disorder	3	(11.1%)
Eating disorders	2	(7.4%)
Anorexia	1	(3.7%)
Bulimia	1	(3.7%)
Nonorganic insomnia	2	(7.4%)

Based on these diagnostic assessments, four types of treatment recommendations were issued. The distribution of recommendations is shown in Table 3.

**Table 3:** Types of treatment recommendations issued following psychosomatic consultation and their uptake, as assessed at three-month follow-up

Referral to further therapy (*n* = 27 interviews)	*n*	received as recommended (proportion achieving %)	continuation of care not achieved (proportion not- achieving %)	no follow-up data
Further psychosomatic/psychiatric consultation	4	4 (100.0%)	0 (0.0%)	0
Inpatient specialised psychosomatic treatment	7	2 (28.6%)	2 (28.6%)	3
Outpatient psychotherapy	5	2 (40.0%)	1 (20.0%)	2
Psychosocial counseling center (f.e. regarding addiction)	3	1 (33.3%)	1 (33.3%)	1

At three-month follow-up, treatment uptake varied across recommendation types. Six of the 19 patients (31.6%) with confirmed diagnoses could not be reached for follow-up assessment. Among the remaining patients, uptake differed substantially depending on the recommended treatment pathway. Complete follow-up data and recommendation-specific uptake rates are presented in Table 3.

### Screening-initiated vs. clinician-initiated referrals - comparative outcomes

Referral rates for psychosomatic consultation differed substantially between study conditions. On the intervention ward, where systematic PHQ-4 screening was implemented, 21 of 127 patients (16.5%) were referred for consultation. In contrast, on the control wards operating under treatment-as-usual procedures, only 22 of 267 patients (8.2%) received a psychosomatic referral (*p*=0.02, Fisher’s exact test). While psychiatric consultations were requested for a small proportion of patients in both groups (intervention: *n* = 3, 2.9%; control: *n* = 12, 4.5%), these were not included in the primary analyses. Exclusion was necessary due to the absence of structured diagnostic interviews and insufficient documentation to establish temporal attribution relative to the screening intervention and usual care referral processes.

When psychosomatic and psychiatric referrals are considered jointly, the overall rate of any mental-health–related referral was still numerically higher in the intervention condition than in the control condition. On the intervention ward, 24 of 127 patients (18.9%) received either a psychosomatic or psychiatric referral. On the control wards, the corresponding rate was 34 of 267 patients (12.7%) (*p* = 0.13, Fisher’s exact test).

We then compared the two referral pathways—systematic screening (intervention) initiating a referral to psychosomatic consultation versus medical staff referral (treatment as usual) in the control group — for three key outcomes: lag time between admission and referral for consultation, lag time between admission and consultation, and diagnostic confirmation (Mini-DIPS) among referred patients.

There was no statistically significant difference in time from admission to referral between the control and intervention groups (median 2.5 vs. 1.0 days; Mann–Whitney U = 113.0, *p* = 0.30). However, time from admission to consultation was significantly longer in the control group, with patients waiting approximately 2.5 days longer for consultation (median 3.5 vs. 1.0 days; Mann–Whitney U = 140.5, *p* = 0.01). Medians are reported due to a statistical outlier in the control group: one patient was not referred until day 62 of admission (see Table 1).

**Fig. 2:**
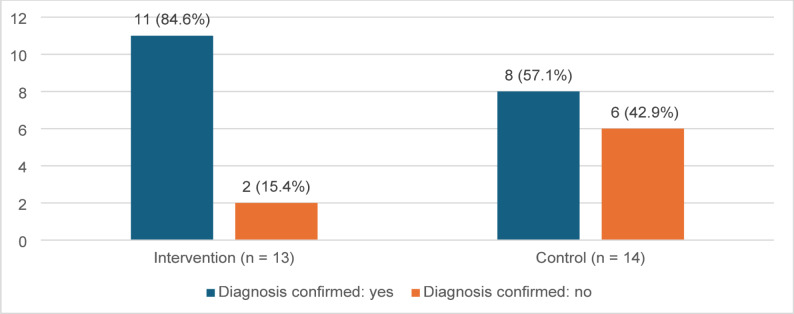
Confirmed psychiatric diagnoses after referral in the intervention ward and control wards. Note Bars represent the proportion of referred patients with and without a confirmed psychiatric diagnosis based on the structured clinical interview (Mini-DIPS). The intervention group represents referrals following PHQ-4 screening, whereas the control group represents clinician-initiated referrals

Screening-based referrals showed higher diagnostic yield. Among intervention referrals, 11 of 13 consultations (84.6%) confirmed clinically relevant psychiatric disorders, compared with 8 of 14 control referrals (57.1%) (*p* = 0.21, Fisher’s exact test) (Fig. 2). This difference did not reach statistical significance.

## Discussion

This pilot study examined whether systematic PHQ-4 screening may improve the identification of clinically relevant psychiatric comorbidities in general hospital inpatients compared with usual clinician-initiated referral for psychosomatic CL-service. We aimed to compare both referral pathways regarding diagnostic confirmation rate, referral and consultation timing, and overall consultation rates, to explore whether structured screening may offer advantages in early detection and timely psychosomatic care.

Systematic PHQ-4 screening was associated with a numerically higher proportion of patients with validated psychiatric diagnoses compared with referral based solely on medical staff judgement, as well as shorter time from admission to consultation. While the difference in diagnostic confirmation did not reach statistical significance, these findings suggest that systematic screening may facilitate earlier and more targeted identification of clinically relevant psychiatric comorbidity in routine hospital care.

Overall, screening identified psychiatric diagnoses in a larger proportion of admitted patients (8.7%) than treatment-as-usual referral (3.0%). These proportions reflect the share of admissions in which a psychiatric disorder was confirmed by structured diagnostic interview within the referral process. Published prevalence estimates indicate that psychiatric comorbidity is common among general medical and surgical inpatients, with depression and anxiety disorders affecting a substantial proportion of patients [1, 2]. However, both identification strategies likely underestimate the true prevalence of psychiatric comorbidity, as structured diagnostic interviews were conducted only in referred patients.

Systematic PHQ-4 screening also doubled the frequency of psychosomatic consultations compared with TAU- referral. Even when psychiatric consultations were added to the respective ward-level totals, overall mental-health–related referral rates remained numerically higher in the intervention condition. Similar effects have been reported in recent studies, with systematic electronic medical record screening increasing consultation rates up to threefold [5]. Combined with the higher diagnostic confirmation rate observed in our study, these findings suggest that reliance on clinician-initiated referrals alone may leave a substantial proportion of psychiatric comorbidities undetected and untreated. While the present study does not directly assess underlying morbidity in non-referred patients, this interpretation aligns with previous evidence indicating that traditional referral pathways under-identify mental health disorders in medical inpatients [7], reinforcing recommendations for routine screening in medical settings [22].

Screening-based referral was associated with shorter time to psychosomatic consultation compared with treatment-as-usual, although this finding should be interpreted cautiously due to a statistical outlier in the control group. The study design may have contributed to shorter lag times from admission to consultation in the intervention group, as research staff presence during screening could have facilitated prompt consultations. The more robust process indicator was time from admission to referral, which was numerically shorter (median difference 1.5 days) in the intervention group, although this difference did not reach statistical significance. The observed pattern is consistent with findings from proactive consultation-liaison service models [23, 24] and may support the potential role of systematic screening in facilitating earlier psychosocial involvement in general hospital settings. However, these findings must be interpreted in the context of the identification strategy. Screening was performed at admission, whereas clinician-initiated referrals may occur later during hospitalization as psychiatric symptoms may emerge over time. Thus, differences in timing could partly reflect structural aspects of the referral process rather than true differences in responsiveness.

At the same time, the lower diagnostic confirmation rate among clinician-initiated referrals suggests that referral decisions in routine care may also be influenced by factors beyond clearly identifiable psychiatric morbidity, such as general psychosocial concerns or patient requests. In this context, systematic screening may contribute to a more structured and potentially more targeted identification process.

Median length of stay differed between groups, with patients on the screening ward showing a longer median hospital stay (5 vs. 3 days). Because the study used a non-randomized ward-based design, this difference may reflect variations in patient case-mix or ward-specific treatment patterns rather than an effect of the screening procedure itself. Consequently, no conclusions can be drawn regarding the relationship between systematic screening and hospital length of stay. Future studies using controlled designs are needed to determine whether systematic screening and earlier psychosocial involvement influence hospital length of stay more generally.

Interestingly, our findings are compatible with the idea that prolonged hospitalization may function as a trigger for psychosomatic consultation requests by medical staff, as patients with the longest length of stay were disproportionately likely to be referred. An association between time to referral and length of stay has been reported previously [25]. These findings raise the possibility that earlier identification of psychosocial needs may help address such delays in consultation and potentially improve care pathways.

A potentially higher detection rate of mental disorders—and the resulting increase in psychosomatic consultation referrals—must be balanced against the clinical reality of limited resources. In practice, the majority of patients cannot receive mental health co-treatment, and an effective psychiatric or psychosomatic C-L service must prioritize those with the greatest needs. This raises the clinically important question: Which patients should receive C-L- consultations?

From our perspective, the presence of clinically relevant psychiatric comorbidity represents the most important criterion. However, the reasons given by medical staff for referral highlight additional relevant factors, such as explicit patient requests or clearly identifiable psychosocial stressors (e.g., recent bereavement or job loss). This points to the need for structured criteria to define psychosocial burdens that warrant intervention. One example is the INTERMED approach, which systematically assesses biopsychosocial health risks and needs [26].

Precise identification of patients requiring intervention is only one component of integrated care. In our study, follow-up revealed limited uptake of recommended treatments—particularly inpatient care—which underscores the need to simplify access pathways. Screening can contribute to faster and more accurate detection, but it must be accompanied by timely treatment capacity, simplified referral processes, and potentially specialized settings for patients with physical illness [27]. In addition, closer coordination between consultation–liaison services and follow-up providers, as well as clearer communication of referral options to patients, may help facilitate access to recommended treatments. As Mitchell et al. [3] emphasize, screening tools are only useful if coupled with adequate follow-up and treatment. Without reducing barriers to care, screening may be potentially frustrating and not productive.

From a health systems perspective, systematic screening may also have implications for resource allocation. In the present study, clinician-initiated referrals resulted in a lower proportion of confirmed psychiatric diagnoses compared with screening-based referrals, suggesting that consultation resources were partly allocated to patients without a confirmed mental disorder. More targeted identification through screening could therefore contribute to a more efficient use of consultation–liaison services by prioritizing patients with a higher likelihood of clinically relevant psychiatric comorbidity.

### Limitations

This study has several important limitations. First, its pilot nature and small sample size limit the statistical power to detect effects and the robustness of subgroup analyses. Second, the non-randomized design, with separate wards allocated to intervention and control conditions, introduces risk of selection bias and confounding by ward-specific factors, including staff attitudes, patient characteristics, and organizational culture.

Third, there was no blinding, as the same authors conducted both screening and diagnostic interviews, which may have introduced observer bias.

Fourth, structured diagnostic interviews were performed only in patients referred for psychosomatic consultation and not in the entire inpatient population. Consequently, the true prevalence of psychiatric disorders in the sample remains unknown, and false-negative cases among screening-negative or non-referred patients could not be identified. This also precluded calculation of sensitivity and specificity for the screening approach. Dropout analysis indicated that interviewed patients were younger on average than those not interviewed, suggesting that older or less fit patients were less likely to participate despite potential indications for psychosomatic or psychiatric co-treatment.

In addition, not all referred patients completed diagnostic interviews, follow-up data were incomplete for some patients, and detailed reasons for non-uptake of recommended treatments were not systematically recorded in this pilot study, which limits interpretation of treatment uptake.

Fifth, the study was conducted in a gastroenterological inpatient population, in which psychiatric comorbidity is known to be relatively prevalent [28, 29]. As prevalence influences the predictive value of screening approaches, the observed diagnostic confirmation rates may be specific to this clinical setting and should be generalized with caution.

Finally, psychiatric consultations occurred alongside psychosomatic services during the study period. In routine clinical practice, referring physicians may choose between psychosomatic and psychiatric consultation services depending on their clinical judgement. Given the overlap between conditions managed by both services (e.g., affective disorders presenting with varying severity), this may have influenced referral patterns in the present study and represents an additional source of variability in consultation pathways. Because the psychiatric consultations did not include standardized diagnostic documentation and their temporal relationship to psychosomatic consultations could not be reliably established, they were reported descriptively and not included in the primary analyses. In addition, the distinction between psychosomatic and psychiatric consultation services reflects the specific organization of the German healthcare system and may limit the generalizability of these findings to other settings.

## Conclusions

Systematic PHQ-4 screening was associated with a numerically higher confirmation of psychiatric comorbidities and earlier psychosomatic consultation compared with clinician-initiated referral. These findings suggest that reliance on routine referral alone may leave a substantial proportion of psychiatric morbidity undetected in general hospital settings. However, given the quasi-experimental design and limited sample size, the results should be interpreted cautiously. Larger randomized studies are needed to determine whether systematic screening improves patient outcomes and how it can be effectively integrated into routine hospital workflows. Evidence from such studies could inform national and international recommendations for the early identification of mental disorders in somatic care.

## Data Availability

The data that support the findings of this study are available from the corresponding author upon reasonable request. Due to ethical restrictions and patient confidentiality, raw data cannot be made publicly available.

## Abbreviations

GAD: 7 Generalized Anxiety Disorder-7
GDS: Geriatric Depression Scale
GHQ: General Health Questionnaire
ICD: 10-International Classification of Diseases
MINI: DIPS-Short Diagnostic Interview for Mental Disorders
PHQ: Patient Health Questionnaire
TAU: Treatment as usual
